# Vascular consequences of passive Aβ immunization for Alzheimer's disease. Is avoidance of "malactivation" of microglia enough?

**DOI:** 10.1186/1742-2094-2-2

**Published:** 2005-01-11

**Authors:** Steven W Barger

**Affiliations:** 1 Department of Geriatrics, University of Arkansas for Medical Sciences, Little Rock, Arkansas 72205 USA; 2 Department of Neurobiology & Developmental Sciences, University of Arkansas for Medical Sciences, Little Rock, Arkansas 72205 USA; 3 Geriatric Research Education and Clinical Center, Central Arkansas Veterans Healthcare System, Little Rock, Arkansas 72205 USA

## Abstract

The role of inflammation in Alzheimer's disease (AD) has been controversial since its first consideration. As with most instances of neuroinflammation, the possibility must be considered that activation of glia and cytokine networks in AD arises merely as a reaction to neurodegeneration. Active, healthy neurons produce signals that suppress inflammatory events, and dying neurons activate phagocytic responses in microglia at the very least. But simultaneous with the arrival of a more complex view of microglia, evidence that inflammation plays a causal or exacerbating role in AD etiology has been boosted by genetic, physiological, and epidemiological studies. In the end, it may be that the semantics of "inflammation" and glial "activation" must be regarded as too simplistic for the advancement of our understanding in this regard. It is clear that elaboration of the entire repertoire of activated microglia – a phenomenon that may be termed "malactivation" – must be prevented for healthy brain structure and function. Nevertheless, recent studies have suggested that phagocytosis of Aβ by microglia plays an important role in clearance of amyloid plaques, a process boosted by immunization paradigms. To the extent that this clearance might produce clinical improvements (still an open question), this relationship thus obligates a more nuanced consideration of the factors that indicate and control the various activities of microglia and other components of neuroinflammation.

## Introduction

Alzheimer's disease (AD) is a progressive degeneration of neural structure and function that arises in the cerebral cortex. Behaviorally, affected individuals usually present with semantic difficulty, followed by a deficiency in episodic memory, spatial disorientation, sleep disturbances, depression, agitation, loss of longer memories, general difficulty with the activities of daily living, and eventually, death. Neuropathological findings include a relatively high number of extracellular deposits of the amyloid β-peptide (Aβ), argyrophillic cytoskeletal aggregates in neurons, accumulation of α-synuclein, loss of synapses, loss of cholinergic and adrenergic fibers, loss of pyramidal neurons, and cerebral amyloid angiopathy (CAA) – deposition of Aβ around blood vessels.

Most of the AD correlates above have been connected in some way to inflammation. For instance, the plaques – comprised primarily of aggregated amyloid β-peptide (Aβ) – are inundated with microglia that show profiles of morphology and gene expression consistent with inflammation. Indeed, if one characterizes any activity by microglia as a sign of "neuroinflammation," it can be said that inflammatory responses have been evident in AD for at least 40 years [1]. But, it was not until the late 1980s that investigators were willing to express the hypothesis that inflammatory events were causal or otherwise contributing to the progression of the disease. Recognition of the powerful impact of a cytokine like interleukin-1 (IL-1), elevated in AD microglia, permitted such speculation [2]. Similarly, research accrued showing that primary inflammation could lead to many of the aberrations found in AD, fueling the consideration that inflammatory events were seminal [3-5]. Many of the individual molecules produced by activated microglia and astrocytes are conditional neurotoxins: hydrogen peroxide, glutamate and other agonists of glutamate receptors, complement components, prostanoids. (Nitric oxide from inducible nitric oxide synthase, produced abundantly in rodent glia, may be less important in human tissues.) Retrospective epidemiological studies showed protection against AD – either in age of onset or rate of progression – by nonsteroidal antiinflammatory drugs (NSAIDs); such correlations have now been born out in a prospective study [6]. Perhaps most compelling, polymorphisms in the genes for proinflammatory cytokines are indicative of risk for AD [7].

Despite these indications, there are reasons to believe that the changes observed in glia and inflammatory cytokines constitute a compensatory response in AD. Indeed, some investigators have been reluctant to apply the term "inflammation" to the constellation of events related to AD pathology. Some of the cytokines and other gene products expressed in peripheral sites of inflammation are present in the AD brain, but there is no apparent vasodilation or extravasation of neutrophils. In general, there seems to be less of the molecular and cellular behavior that is responsible for bystander tissue damage in peripheral inflammation. This journal was founded partially out of recognition that "neuroinflammation" is distinct. In essence, the concept reflects a compromise befitting the difficult line that must be maintained between effective cell-mediated immune responses and damage to the precious components of the CNS. Microglia elevate their expression of neurotrophic factors under many of the same conditions in which they show inflammation-related responses such as phagocytosis, retraction of processes, release of excitotoxins, and production of IL-1β and IL-6 and tumor necrosis factor [8]; in fact, the latter cytokines can have neurotrophic effects themselves [9,10]. Astrocytes deposit proteoglycans around the Aβ deposits destined to become plaques [11], perhaps sequestering this neurotoxic peptide from doing its harm. Even the apparent benefits of NSAIDs can be parsed from their presumed mechanism of inhibiting cyclooxygenase-2 [12,13](and references therein).

## Discussion

Recent experiments with anti-Aβ immunization have highlighted another beneficial effect of "activated" microglia: removal of Aβ. It has long been recognized that microglia can efficiently phagocytose and at least partially degrade Aβ both in vitro and in vivo. But the persistence of amyloid plaques suggests that microglia are stymied in this regard during the development of AD or in the deposition of Aβ in mice transgenically engineered to produce large amounts of the peptide. Introduction of antibodies recognizing Aβ, either by active vaccination or by passive immunization (injection of antibodies, typically monoclonal), results in removal of some Aβ deposits and/or prevention of their formation. Although the phenomenon has been studied most rigorously in the transgenic mouse models, similar clearance of parenchymal plaques seems to have occurred in two human subjects that participated in an Aβ-vaccine trial [14,15]. And microglia appear to contribute; Aβ can be readily detected in microglia of immunized mice [16] and was also abundant in some microglia and related syncitia in the AD trial subjects [14,15]. However, the only reason we are privy to the effects of the vaccination paradigm in humans is because these two individuals died after complications of meningeal encephalitis – rampant cranial inflammation brought on by the immunization. This iatrogenic event occurred in about five percent of the human subjects vaccinated against Aβ, prompting discontinuation. One interesting finding from both autopsies is that while parenchymal Aβ deposits were substantially lower than to be expected in AD victims, both individuals had relatively high levels of vascular deposition. This CAA was accompanied by microhemorrhage in at least one of the subjects [15], consistent with the majority of advanced cases of CAA [17].

Wilcock *et al*. [18] have now produced evidence that the appearance of CAA after immunization may represent an actual increase in this parameter triggered by anti-Aβ antibodies. Furthermore, the investigators also found that the CAA was accompanied by an increase in hemorrhages – similar to a previous report [19] – and a vascular accumulation of CD45^+ ^cells presumed to be microglia. The experimental paradigm was one of passive immunization of transgenic mice at nearly two years of age, old enough to have accumulated substantial Aβ deposits. Consistent with expectations, injection of anti-Aβ antibody diminished deposits in the parenchyma, even those that were mature enough to stain with Congo red. However, vascular deposition of Congo-red staining was elevated by approximately four-fold in the anti-Aβ-treated animals. Pfeiffer *et al*. found similar results in another transgenic line [19]. Further, Wilcock *et al*. now show that the regional accumulation of vascular amyloid was accompanied by an elevated index of hemorrhages and a congregation of CD45^+ ^cells, presumed to be microglia [18]. Given that stromal microglia show increased signs of activity and contain Aβ after passive Aβ immunization [20], one interpretation is that the immunization-induced shift in amyloid from the parenchyma to the vasculature is mediated by phagocytic microglia attempting to discard the Aβ into the bloodstream. Such a phenomenon is tenuously supported by the analogous transport of pyknotic neuronal nuclei to the vasculature by microglia, observed in 3-D time-lapse videos by Dailey and coworkers [21]. In those images, microglia are occasionally seen to transfer the nuclei to another cell, conceivably a perivascular macrophage or dendritic cell. Thus, it is not clear whether the CD45^+ ^cells observed by Wilcock and coworkers are microglia or another cell type. It is also unclear whether the accumulation of amyloid and inflammatory cells at the blood vessels represents an arrested state in Aβ clearance or simply a bottleneck in the transport, one that would eventually yield to complete removal of the peptide. However, the appearance of CAA in the human subjects that suffered from acute encephalitis suggests that the vascular accumulation is an untoward event, created or facilitated by inflammation. Another vascular irregularity caused by Aβ has been linked to inflammatory events in both transgenic mice and isolated human blood vessels [22].

The apparent contributions of inflammatory mechanisms to both Aβ clearance and vascular pathology illustrate a somewhat unique example of microglial ambivalence. While many had argued that microglial "activation" by Aβ was at least partially responsible for AD-associated degeneration, others had pointed to microglial phagocytosis as a desirable consequence of activation. For the purposes of discussion, the term "*mal*activation" will be applied here to microglial activation which produces neurodegeneration. One obvious question is whether there might be a mode of "activation" that permits phagocytosis while limiting malactivation. In fact, stimulation of Fc receptors – the antibody receptors that initiate a good deal of antibody-triggered phagocytosis – can inhibit cytotoxicity in macrophages [23]. Similarly, phagocytosis of apoptotic cells inhibits macrophage expression of proinflammatory cytokines like IL-1, IL-8, tumor necrosis factor, and several prostanoids through stimulation of a phosphatidylserine receptor [24]. Evidence indicates that malactivation involves the production of reactive oxygen species like superoxide and peroxide, nitric oxide, and excitotoxins (glutamate, quinolinate, and D-serine). If these criteria are germane, malactivation certainly can be suppressed by specific cytokines, such as transforming growth factor β (TGFβ) [25]. Although TGFβ has often been characterized broadly as "anti-inflammatory," it does not inhibit the phagocytic activity of microglia in a setting where another "anti-inflammatory" cytokine (IL-4) does [26]. Interestingly, TGFβ1 transgenesis promotes the same apparent shift of Aβ from parenchyma to vessel that is observed after Aβ immunization [27].

## Conclusions

While some have argued that CAA is of little consequence in AD [28], the elaboration of the deposition that appears to occur under conditions of "beneficial inflammation" is on par with that seen in hereditary cerebral hemorrhage with angiopathy-Dutch type and is certainly a risk factor for devastating levels of hemorrhage. If such a response reflects a broad-acting realignment of cytokine profiles contingent upon immunization, it behooves careful consideration (and extensive animal testing) for any strategy for antibody-mediated reduction of Aβ in the AD brain.

## List of abbreviations

AD: Alzheimer's disease

Aβ: amyloid β-peptide

CAA: cerebral amyloid angiopathy

IL-1, -6, -8: interleukin-1, -6, -8

NSAID: nonsteroidal antiinflammatory drug

TGFβ: transforming growth factor β

## Competing interests

The author(s) declare that they have no competing interests.

## References

[B1] Terry RD, Gonatas NK, Weiss M (1964). Ultrastructural studies in Alzheimer's presenile dementia.. Am J Pathol.

[B2] Griffin WST, Stanley LC, Ling C, White L, MacLeod V, Perrot LJ, White CLIII, Araoz C (1989). Brain interleukin 1 and S-100 immunoreactivity are elevated in Down syndrome and Alzheimer disease. Proc Natl Acad Sci USA.

[B3] Willard LB, Hauss-Wegrzyniak B, Danysz W, Wenk GL (2000). The cytotoxicity of chronic neuroinflammation upon basal forebrain cholinergic neurons of rats can be attenuated by glutamatergic antagonism or cyclooxygenase-2 inhibition. Exp Brain Res.

[B4] Li Y, Liu L, Barger SW, Griffin WS (2003). Interleukin-1 mediates pathological effects of microglia on tau phosphorylation and on synaptophysin synthesis in cortical neurons through a p38-MAPK pathway. J Neurosci.

[B5] Craft JM, Van Eldik LJ, Zasadzki M, Hu W, Watterson DM (2004). Aminopyridazines attenuate hippocampus-dependent behavioral deficits induced by human beta-amyloid in a murine model of neuroinflammation. J Mol Neurosci.

[B6] Zandi PP, Anthony JC, Hayden KM, Mehta K, Mayer L, Breitner JC (2002). Reduced incidence of AD with NSAID but not H2 receptor antagonists: the Cache County Study. Neurology.

[B7] Mrak RE, Griffin WS (2000). Interleukin-1 and the immunogenetics of Alzheimer disease. J Neuropathol Exp Neurol.

[B8] Elkabes S, DiCicco-Bloom EM, Black IB (1996). Brain microglia/macrophages express neurotrophins that selectively regulate microglial proliferation and function. J Neurosci.

[B9] Hama T, Kushima Y, Miyamoto M, Kubota M, Takei N, Hatanaka H (1991). Interleukin-6 improves the survival of mesencephalic catecholaminergic and septal cholinergic neurons from postnatal, two-week-old rats in cultures. Neuroscience.

[B10] Barger SW, Hörster D, Furukawa K, Goodman Y, Kriegelstein J, Mattson MP (1995). Tumor necrosis factors a and b protect neurons against amyloid b-peptide toxicity:  Evidence for involvement of a kB-binding factor and attenuation of peroxide and Ca2+ accumulation. Proc Natl Acad Sci USA.

[B11] Shioi J, Pangalos MN, Ripellino JA, Vassilacopoulou D, Mytilineou C, Margolis RU, Robakis NK (1995). The Alzheimer amyloid precursor proteoglycan (appican) is present in brain and is produced by astrocytes but not by neurons in primary neural cultures. J Biol Chem.

[B12] Aisen PS, Schafer KA, Grundman M, Pfeiffer E, Sano M, Davis KL, Farlow MR, Jin S, Thomas RG, Thal LJ (2003). Effects of rofecoxib or naproxen vs placebo on Alzheimer disease progression: a randomized controlled trial. Jama.

[B13] Weggen S, Eriksen JL, Sagi SA, Pietrzik CU, Ozols V, Fauq A, Golde TE, Koo EH (2003). Evidence that nonsteroidal anti-inflammatory drugs decrease amyloid beta 42 production by direct modulation of gamma-secretase activity. J Biol Chem.

[B14] Nicoll JA, Wilkinson D, Holmes C, Steart P, Markham H, Weller RO (2003). Neuropathology of human Alzheimer disease after immunization with amyloid-beta peptide: a case report. Nat Med.

[B15] Ferrer I, Boada Rovira M, Sanchez Guerra ML, Rey MJ, Costa-Jussa F (2004). Neuropathology and pathogenesis of encephalitis following amyloid-beta immunization in Alzheimer's disease. Brain Pathol.

[B16] Games D, Bard F, Grajeda H, Guido T, Khan K, Soriano F, Vasquez N, Wehner N, Johnson-Wood K, Yednock T, Seubert P, Schenk D (2000). Prevention and reduction of AD-type pathology in PDAPP mice immunized with A beta 1-42. Ann N Y Acad Sci.

[B17] Pfeifer LA, White LR, Ross GW, Petrovitch H, Launer LJ (2002). Cerebral amyloid angiopathy and cognitive function: the HAAS autopsy study. Neurology.

[B18] Wilcock DM, Rojiani A, Rosenthal A, Subbarao S, Freeman MJ, Gordon MN, Morgan D (2004). Passive immunotherapy against Abeta in aged APP-transgenic mice reverses cognitive deficits and depletes parenchymal amyloid deposits in spite of increased vascular amyloid and microhemorrhage. J Neuroinflammation.

[B19] Pfeifer M, Boncristiano S, Bondolfi L, Stalder A, Deller T, Staufenbiel M, Mathews PM, Jucker M (2002). Cerebral hemorrhage after passive anti-Abeta immunotherapy. Science.

[B20] Wilcock DM, Rojiani A, Rosenthal A, Levkowitz G, Subbarao S, Alamed J, Wilson D, Wilson N, Freeman MJ, Gordon MN, Morgan D (2004). Passive amyloid immunotherapy clears amyloid and transiently activates microglia in a transgenic mouse model of amyloid deposition. J Neurosci.

[B21] Petersen MA, Dailey ME (2004). Diverse microglial motility behaviors during clearance of dead cells in hippocampal slices. Glia.

[B22] Paris D, Humphrey J, Quadros A, Patel N, Crescentini R, Crawford F, Mullan M (2003). Vasoactive effects of A beta in isolated human cerebrovessels and in a transgenic mouse model of Alzheimer's disease: role of inflammation. Neurol Res.

[B23] Virgin HW, Kurt-Jones EA, Wittenberg GF, Unanue ER (1985). Immune complex effects on murine macrophages. II. Immune complex effects on activated macrophages cytotoxicity, membrane IL 1, and antigen presentation. J Immunol.

[B24] Fadok VA, Bratton DL, Rose DM, Pearson A, Ezekewitz RA, Henson PM (2000). A receptor for phosphatidylserine-specific clearance of apoptotic cells. Nature.

[B25] Suzumura A, Sawada M, Yamamoto H, Marunouchi T (1993). Transforming growth factor-beta suppresses activation and proliferation of microglia in vitro. J Immunol.

[B26] Chan A, Magnus T, Gold R (2001). Phagocytosis of apoptotic inflammatory cells by microglia and modulation by different cytokines: mechanism for removal of apoptotic cells in the inflamed nervous system. Glia.

[B27] Wyss-Coray T, Lin C, Yan F, Yu GQ, Rohde M, McConlogue L, Masliah E, Mucke L (2001). TGF-beta1 promotes microglial amyloid-beta clearance and reduces plaque burden in transgenic mice. Nat Med.

[B28] Castellani RJ, Smith MA, Perry G, Friedland RP (2004). Cerebral amyloid angiopathy: major contributor or decorative response to Alzheimer's disease pathogenesis. Neurobiol Aging.

